# The performance of field sampling for parasite detection in a wild passerine

**DOI:** 10.1002/ece3.9242

**Published:** 2022-08-23

**Authors:** Salamatu Abdu, Michael Chimento, Gustavo Alarcón‐Nieto, Daniel Zúñiga, Lucy M. Aplin, Damien R. Farine, Hanja B. Brandl

**Affiliations:** ^1^ Department of Biology University of Konstanz Constance Germany; ^2^ Department of Collective Behaviour Max Planck Institute of Animal Behavior Radolfzell Germany; ^3^ Department of Evolutionary Biology and Environmental Studies University of Zurich Zurich Switzerland; ^4^ Centre for the Advanced Study of Collective Behaviour Universität Konstanz Constance Germany; ^5^ Cognitive and Cultural Ecology Research Group Max Planck Institute of Animal Behavior Radolfzell Germany; ^6^ Division of Ecology and Evolution, Research School of Biology Australian National University Canberra Australian Capital Territory Australia

**Keywords:** fecal egg count, field sampling, McMaster, mini‐FLOTAC, parasite infection, repeatability

## Abstract

Parasites can impact the behavior of animals and alter the interplay with ecological factors in their environment. Studying the effects that parasites have on animals thus requires accurate estimates of infections in individuals. However, quantifying parasites can be challenging due to several factors. Laboratory techniques, physiological fluctuations, methodological constraints, and environmental influences can introduce measurement errors, in particular when screening individuals in the wild. These issues are pervasive in ecological studies where it is common to sample study subjects only once. Such factors should be carefully considered when choosing a sampling strategy, yet presently there is little guidance covering the major sources of error. In this study, we estimate the reliability and sensitivity of different sampling practices at detecting two internal parasites—*Serratospiculoides amaculata* and *Isospora* sp.—in a model organism, the great tit *Parus major*. We combine field and captive sampling to assess whether individual parasite infection status and load can be estimated from single field samples, using different laboratory techniques—McMaster and mini‐FLOTAC. We test whether they vary in their performance, and quantify how sample processing affects parasite detection rates. We found that single field samples had elevated rates of false negatives. By contrast, samples collected from captivity over 24 h were highly reliable (few false negatives) and accurate (repeatable in the intensity of infection). In terms of methods, we found that the McMaster technique provided more repeatable estimates than the mini‐FLOTAC for *S. amaculata* eggs, and both techniques were largely equally suitable for *Isospora* oocysts. Our study shows that field samples are likely to be unreliable in accurately detecting the presence of parasites and, in particular, for estimating parasite loads in songbirds. We highlight important considerations for those designing host–parasite studies in captive or wild systems giving guidance that can help select suitable methods, minimize biases, and acknowledge possible limitations.

## Graphical abstract

Quantifying endoparasites in wild animals can be challenging and often opportunistic. In our study, we show that pooled samples while holding animals in captivity can improve parasite detection.

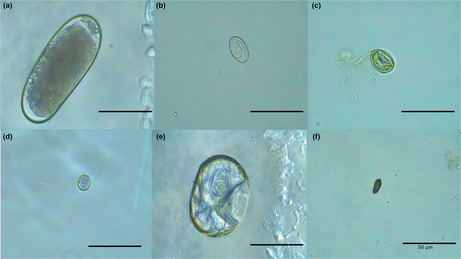

## 1 INTRODUCTION

Parasites exist in diverse forms and infect a wide range of taxa, often affecting the behavior, fitness, and ecology of their hosts (Ferreira et al., 2019). For example, recent studies have found that parasites can alter individuals' activity levels (Chapman et al., 2016), interactions with conspecifics (Xu et al., 2021), movement (Jolles et al., 2020), and survival (Brown & Brown, 1986; Jolles et al., 2008). Advances in bio‐logging (Whitford & Klimley, 2019) have further opened up opportunities to study fine‐scale behaviors and interactions in free‐living animals alongside physiological parameters (e.g., heart rate and body temperature) that could be modulated by parasites. However, understanding the consequences of parasites on ecology and behavior in the wild typically relies on collecting field samples from individuals (Dib et al., 2020). These samples, often obtained at a single time point, are typically assumed to capture individuals' true infection state, but this assumption is rarely tested (Miller et al., 2018). Accurate estimates of infection state are particularly critical when studying the consequences of parasites on outcomes such as changes in behavior attributed to environmental factors and interactions between conspecifics, because infection state is often used as a predictor and most modeling approaches assume that estimates of predictors are error‐free. Thus, validating the accuracy of estimates of endoparasite infection state, or load, estimated from field data is important.

Collecting data on endoparasite loads in wild animals is challenging. A common non‐invasive sampling method involves processing feces of animal hosts and counting excreted eggs/oocysts using laboratory techniques (Roepstorff & Nansen, 1998). However, if such samples provide inaccurate estimates of infection status or parasite loads, they could reduce the power to detect biological relationships of interest. Re‐trapping and re‐sampling, where possible, is likely to vastly improve estimates (Knowles et al., 2013), but in most wide‐ranging animals, like many ungulates, it is often only feasible to sample individuals once (Ezenwa et al., 2012). The biases or inaccuracies associated with estimating parasite loads and determining infection state indirectly from feces in the wild therefore cannot be easily eliminated in many systems, but the potential effects of sampling noise are rarely reported or accounted for. Comparative studies explicitly aimed to quantitatively estimate the propensity for different sampling methods to produce noisy estimates of parasite status, can help with both designing and reporting of sampling protocols in field studies.

Several methods are commonly used for detecting parasites. These include molecular techniques, which are very sensitive in detecting specific known parasites, or screening with the aid of a microscope, which can be cheaper and more reliable for broad screening of unknown parasite types and load (Cimino et al., 2015; van Lieshout & Roestenberg, 2015). In vertebrate hosts parasitized by protozoa (single‐celled microscopic organisms) and helminths (parasitic worms), there are two recommended microscopy‐based fecal egg count methods for quantifying parasites shed through feces: the McMaster (Daş et al., 2020; Roepstorff & Nansen, 1998) and mini‐FLOTAC techniques (Cringoli et al., 2010, 2017). The McMaster is widely used for the identification and quantification of parasites from feces (Roepstorff & Nansen, 1998). It is an older and more established technique than the mini‐FLOTAC, with the advantage of being more time efficient. The mini‐FLOTAC, on the other hand, is a newer design, created to be more sensitive and affordable (Cringoli et al., 2010, 2017), but entails a prolonged viewing time. Both techniques have been reported to be reliable (Ballweber et al., 2014), and comparable across several species of host and parasites (Alowanou et al., 2021; Lozano et al., 2021; Silva et al., 2013). However, different parasite species have different properties and may respond differently to these techniques. For example, the McMaster has been reported to be more sensitive than the mini‐FLOTAC technique at detecting some nematode species (Daş et al., 2020; Went et al., 2018). By contrast, the mini‐FLOTAC was suggested to be more sensitive and precise for coccidian oocysts (Lozano et al., 2021; Silva et al., 2013).

Besides the choice of technique, parasite sampling can also be susceptible to variation from intrinsic factors specific to the study design or the parasite of interest (Ballweber et al., 2014). The first source of variation is when samples are collected. The shedding of coccidia oocysts in passerines is influenced by a circadian rhythm, with greater numbers observed in the afternoon (Brawner III & Hill, 1999; López et al., 2007; Villanúa et al., 2006). The second source of variation is the duration of storage, which could degrade parasite eggs (Crawley et al., 2016). Third, the accuracy of parasite counts can be impacted by the amount of sample collected, and while most laboratory techniques have established standard weights to be used, some species might pass out far less feces. Fourth, parasites' phenotypical structure differs greatly within and between species and the parasite can fluctuate in their modes of adaptation to their hosts over time, thereby affecting infection loads. For instance, the fecundity and generation time of a typical nematode or coccidian reflect their distinct lifecycles and reproductive strategies (Burrell et al., 2020; Wharton, 1986), which could affect their detectability under different sampling regimes. Finally, extrinsic factors, such as temperature and precipitation, can also favor the proliferation and propagation of parasites. For example, a distinct spring and summer peak in gastrointestinal parasites in wild Soay sheep suggests that parasites evade harsh temperatures and desiccation associated with winter (Sweeny et al., 2021). Considering all these factors, therefore, is important in field studies, especially those that rely on only a single sample per individual. However, to date, there is relatively little guidance on which of these factors are the most important to consider, and when. Such guidance is important because any noise added during sampling means that estimates may not accurately reflect the real infection status of the animal, with consequences for downstream analysis and hypothesis testing (Poulin, 2019).

Here, we assess potential implications of sampling methodology on estimates of infection status and infection intensity, and the robustness of field samples from individuals that are rarely re‐encountered. We combine field and captive sampling to evaluate the reliability and repeatability of single/opportunistic non‐invasive fecal samples taken in the field to estimate parasites. Additionally, we identify the effects of methodological factors, such as weight of feces, latency to process feces, and time of day, on estimates of parasite presence and loads. We then estimate sensitivity and repeatability of the two most commonly used microscopic methods—McMaster and mini‐FLOTAC—to measure parasite loads via count of oocysts and eggs, which are highly resistant immature stages of protozoa and helminths. We use data from great tits *Parus major* that were trapped in the wild and, for the purpose of another experiment (M. Chimento et al., unpublished data), brought temporarily into captivity. The great tit represents an ideal model species because it is among the most frequently studied passerines and hosts numerous parasite species. Further, as with most wild animals, great tits are difficult to target individually in the field, making repeated sampling challenging. Our results give insights into the sources of variation that can arise in the parasite count of a single individual.

## 2 MATERIALS AND METHODS

### 2.1 Study species and sampling

We conducted this study at the Max Planck Institute of Animal Behavior in Radolfzell, Southern Germany, over a period of 8 weeks (October–December 2020). The study area is characterized by a patchwork of farmland and mixed deciduous and coniferous woodland. Great tits are widespread within this region and are less territorial during the non‐breeding season, forming flocks during the winter months (Aplin et al., 2012) that feed on a variety of food, including invertebrates (e.g., spiders, woodlice), berries, seeds and nuts (e.g., beech mast; Balen, 2002; Hogstad, 2015).

We retrieved three distinct fecal samples from individual birds over the course of this study. We targeted great tits at eight catching sites within a 10‐km radius of the institute, capturing birds in mist nets next to bird feeders baited with sunflower seeds. After extracting birds from the mist net, we immediately transferred them into small individual cloth holding bags. At this point, birds usually pass out feces, and so we placed a thin piece of cardboard into the bottom of each bag allowing us to collect these feces. We named these first samples, “field sample”. These same birds were then housed for 2 days in individual cages in a specialized animal housing facility, as part of another experiment (animal ethics permit 35–9185.81/G‐20/100 held by Dr. Lucy Aplin and granted from the Regierungspräsidium Freiburg Az.). During this time, we collected two fecal samples per individual bird, named the “second” and “third” samples or collectively as the “captive samples” across 2 days. The “second sample”, was retrieved 24–27 h post catching (midway through captivity) from each bird, collected from the paper flooring of their cage (which were replaced as part of regular cleaning). For the “third sample”, we repeated the same procedure 46–48 h post catching (after the birds were removed from the cages). Each sample was individually labeled and stored in a preservative (5% formaldehyde) at room temperature until they were examined. The time of day when the sample was collected—morning/afternoon—was noted for every sample, captive samples included due to fresh samples at the time of collection and the weight of feces was recorded in grams. In total, samples were collected from 46 birds.

### 2.2 Parasitology

All fecal samples were collected and processed by SA, thereby eliminating observer differences. The order of field and captive fecal samples for processing was randomized. We employed the modified McMaster technique as described by Roepstorff and Nansen (1998) and combined the mini‐FLOTAC and fill‐FLOTAC techniques as described by Cringoli et al. (2010, 2017). As recommended by Daş et al. (2020), we used a sugar solution with a specific gravity of 1.27 as the flotation medium suitable for the protozoa and helminths of interest. To prepare the sugar solution, 454 g of sugar was dissolved in 355 ml of distilled water and brought to a boil on low heat to prevent caramelization.

For both techniques, the fecal samples were homogenized thoroughly before dilution with water. We used a ratio of 1 g of feces to 14 ml of water for the McMaster technique. The solution was filtered with a cotton gauze into a labeled test tube to reduce the amount of debris before centrifugation. All samples were centrifuged for 5 mins at 161 *g*‐force. After this, the supernatant was discarded leaving the sediment. The sediment was topped up to a 4 ml mark with the sugar solution prepared above, before viewing under the microscope. The mini‐FLOTAC is a derivative of the FLOTAC which is suitable for use in the field. The simplified mini‐FLOTAC technique eliminates the centrifugation step in the FLOTAC. Instead, it utilizes a kit consisting of a collector and a filter. After homogenizing the feces in floatation medium, the suspension is transferred into a cylindrical disc with 24 viewing chambers on either side. The FLOTAC technique has a more robust and slightly complicated setup involving two centrifugation steps and is most appropriately done in the lab as it requires a large volume centrifuge. The FLOTAC apparatus is similar to the mini‐FLOTAC but can accommodate more fecal sample suspension—10 ml—with two separate wells of 5 ml each. For the FLOTAC technique, to incorporate the centrifugation step, we adopted the preparatory steps of the fill‐FLOTAC technique and used the mini‐FLOTAC apparatus for viewing. Next, we diluted 1 g of feces in 10 ml of water and centrifuged similarly as above. Due to feces weighing 1 g or less, we did not use the fill‐FLOTAC device in homogenizing directly with the floatation medium. We followed the steps described in Cringoli et al. (2010) by centrifuging with water before adding the sugar solution (1:10). The new suspension was homogenized before pipetting into the McMaster slide (volume 0.3 ml) and mini‐FLOTAC disc (volume 2 ml). Both apparatus were left to stand for 10 mins before viewing to allow parasite eggs and oocysts to float to the top. Eggs and oocysts were identified and counted with the aid of a binocular microscope (Leica DM500, Heerbrugg, Switzerland). After parasite eggs and oocysts were counted, the respective numbers were multiplied by 25 for the McMaster technique and by 5 for the mini‐FLOTAC technique. The multiplication factor for the mini‐FLOTAC technique was recalculated as 10, if the dilution was 1:20.

**FIGURE 1:**
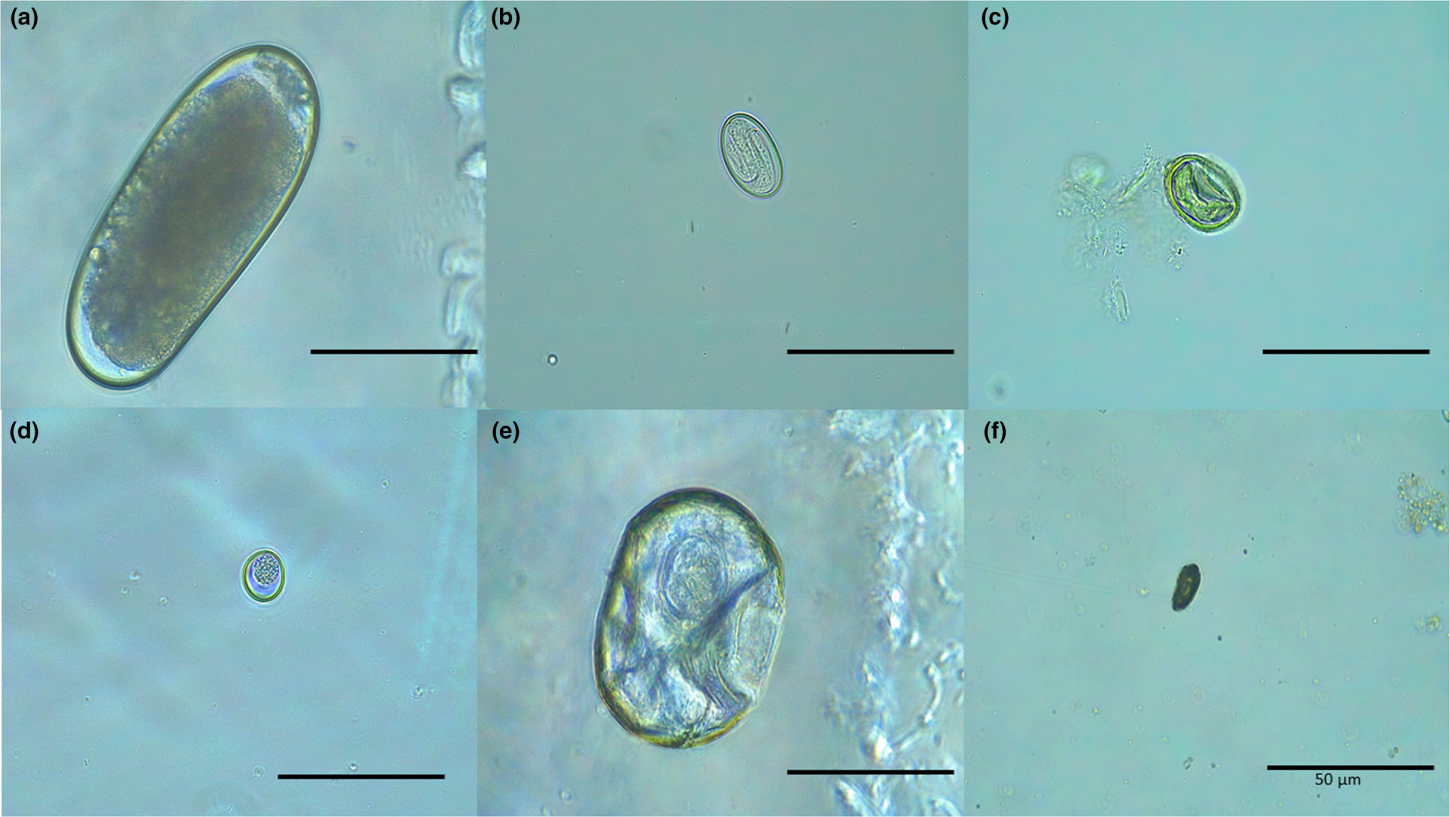
Endoparasites found in the fecal samples of great tits include: (a) an unknown nematode egg, (b) embryonated *S. amaculata* egg, (c) unknown nematode egg, (d) un‐sporulated *Isospora* sp. oocyst (e) *Hymenolepis* sp. egg, and (f) an unknown trematode egg.

Field samples often weighed <1 g and were therefore processed with only one of the two techniques, allocated at random. The second and third samples (i.e., captive samples) were homogenized and split into two halves, with one half processed using the McMaster technique and the other using the mini‐FLOTAC technique. Parasites recovered included coccidia (*Isospora* sp., Figure 1d), trematodes (unidentified, Figure 1f), tapeworms (*Hymenolepis* sp. Figure 1e), and nematodes (air sac worms—*Serratospiculoides amaculata*—Figure 1b and two unidentified spirurids; Figure 1a,c). The load or intensity of infection was described as the egg/oocyst count and the parasites' prevalence was indicated by a presence/absence of infection within an individual bird. Tapeworms, other trematodes, and some nematodes with prevalence of less than 18 percent were excluded from the subsequent analysis as repeatability analyses could not be performed. Coccidia and air sac worms, hereafter, referred to as *Isospora* sp. and *S. amaculata*, respectively, were widely prevalent.

### 2.3 Statistical analysis

All data were analyzed using the R version 3.6.3 (R core team, 2020). Prevalence was calculated as the number of individual birds that were infected with a given parasite divided by the total number of birds sampled. All analyses were conducted identically, but separately, for each parasite type. We then addressed three distinct questions:

#### 2.3.1 How repeatable are field and captive samples across samples and techniques?

Repeatability tests were run to evaluate the consistency of the estimates across samples and techniques, for individual birds using the package “rptR” (Stoffel et al., 2017). Repeatability values were obtained for both infection load and prevalence. We retrieved fecal samples once from 46 individuals in the field. In captivity, we repeated sampling on the same 46 individuals twice, on the second and third days after catching. To compare the McMaster and Mini‐FLOTAC laboratory techniques across the three samples, we used half of the individuals sampled in the field, that is, 23 birds, due to low feces weight as mentioned above. While for captive samples, we used all fecal samples from 46 birds and split each sample in half, therefore summing up to 92 samples per day (46 samples per technique). At the individual level, each bird was sampled thrice, once in the field and twice in captivity.

##### Repeatability between sample types per technique

To estimate the detection repeatability across different sample types, the repeatability of parasite load between field and captive samples, and among captive samples, we used linear mixed models. We fitted these to data from each parasite type, per laboratory techniques—McMaster and mini‐FLOTAC. Repeatability (R) was calculated in each model as the within‐individual variance (how much individual measures varied across each sample analyzed with the same technique) divided by the within‐individual sum of variance and the residual variance (Variance_birdID_/[Variance_birdID_ + Variance_residual_]) (Nakagawa & Schielzeth, 2010). For each parasite, the first two models estimated repeatability between field (first sample) and one captive sample (second sample) for the two techniques separately. Because repeatability was high between captive samples, we chose the second sample as the captive sample for these models (Appendix 1: Table A1, models 1a i, ii and 1b i, ii), while the other two models estimated repeatability between the two captive samples (second and third) for each technique (Appendix 1: Table A1, models 1a iii, iv and 1b iii, iv). We thus included sample number as a fixed categorical variable and bird ID as a random variable (see Appendix 1: Table A1 for all model specifications).

##### Repeatability across techniques

To determine differences in estimates of parasite loads across techniques (McMaster & mini‐FLOTAC), we then ran another repeatability analysis to quantify the across‐technique repeatability. For this, we used technique as a fixed categorical variable, and fit four separate models, one for each combination of parasite type and captive sample number (either the second or third samples, Appendix 1: Table A1, models 2a i, ii and 2b i, ii), and estimated repeatability in each model by dividing the within‐individual variance (how much individual measures varied within each sample when analyzed using one technique or the other) by the within‐individual total variance (Variance_birdID_/[Variance_birdID_ + Variance_residual_]). Bird ID was again added as a random variable.

The eggs/oocysts count per gram for all parasites were log‐transformed to approximate a Gaussian error distribution. We repeated each of the repeatability analyses above with data on prevalence (i.e., infection presence) using binomial generalized mixed models excluding repeatability between first and second samples as values were not obtainable (a singularity error emerged due to the low number of parasite detections between sample types per technique as a result of having fewer field samples per technique). The *R* values were retrieved from the link scale of the model (Appendix 1: Table A1).

#### 2.3.2 What is the probability of detecting false negatives across sample types and techniques?

We calculated the proportion of false negatives for each sample number (i.e., first, second, and third) and lab technique as the proportion of samples from that category in which parasites were not detected that came from the pool of individuals that were otherwise known to be infected. Infected birds were those which returned a positive detection in any of the other samples (irrespective of sample number or technique used).

#### 2.3.3 What factors can influence the detectability of infection status and load?

We used linear mixed models and generalized linear mixed models to measure the effects of sample type, technique, time of day, latency, and weight of feces on the detectability of parasites. We defined latency as the delay in processing samples (calculated as the difference in days between when it was collected and analyzed). Sample type, technique, and time of day were included as categorical variables, while latency and weight of feces were included as continuous variables. Bird ID was added as a random effect. We started with a binomial model fit to presence/absence data using the ‘lme4” package (Bates et al., 2015) to determine the likelihood of detecting parasites. To determine whether the predictors also have an effect on parasite counts, we then subset the data to contain only positive detections, and fit all the variables listed above in a model to test whether they predicted the intensity of infection. We retained all the variables, but compared different models—Poisson distribution and negative binomial distributions (without transformations), log and square root transformations—to find the best‐fitting model based on diagnostic plots from the *performance* R‐package (Lüdecke et al., 2021). For both parasites, we log‐transformed the count data to approximate a Gaussian error distribution, and this gave a better fit than all the other models. We calculated *R* ^2^ values as the sum squared regression multiplied by the total sum of squares subtracted from 1 for each model (Zhang, 2020).

## 3 RESULTS

We sampled 46 birds from eight different sites, of which 11 were older than a year, and 35 were first‐winter birds. Overall, most birds were infected with *Isospora* sp. (80%), while *S. amaculata* was the most prevalent of the nematodes, infecting 37% of individuals. In infected individuals, those with *Isospora* sp. had a median of 220 oocysts per gram (opg; IQR 1050; max.: 128,700 opg), while those with *S. amaculata* had a median egg per gram (epg) load of 288 (epg; IQR 1273; max.: 10,800 epg). Fewer individuals were infected with *Hymenolepis* sp. (tapeworms; 17%; not included in further analyses).

### 3.1 How repeatable are field and captive samples across samples and techniques?

**FIGURE 2:**
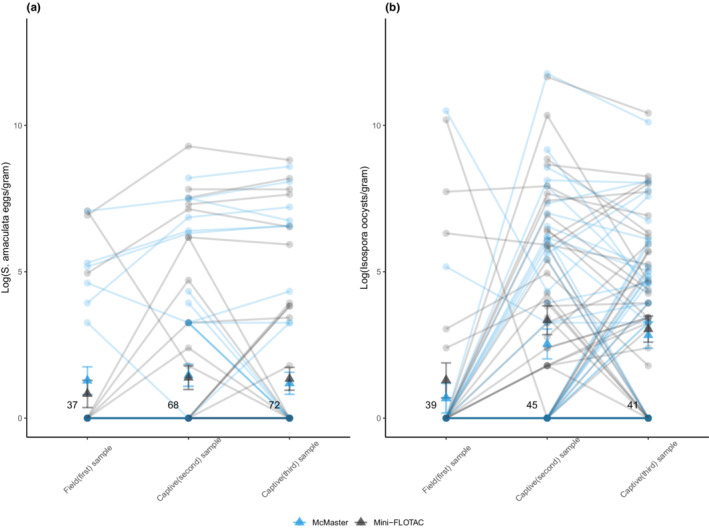
Individual parasite loads across sample types for (a) *S. amaculata* eggs and (b) *Isospora* oocysts. The data are log‐transformed and the lines link parasite loads of the same individual sampled in the field (first) and in captivity (second and third). Field samples were analyzed using only one of the Mini‐FLOTAC (black) or McMaster (blue) techniques, which accounts for fewer lines between the first and second samples (first samples included a total of 46 samples, whereas the second and third samples each included 92 samples). Mean parasite counts and standard errors for each method and sample type are represented by triangular points. Bold numbers represent the number of data points that fall on zero.

Fewer infections were detected in field samples (first sample) than captive samples (second and third) for both *S. amaculata* and *Isospora* sp. (Table 1; Figure 2). We found that field samples detected levels of *S. amaculata* infections of 25 epg and above with less variability across all samples (Figure 2a). For *Isospora* oocysts, however, the field sample failed to reveal many high and low infections, which is apparent by the high proportion of lines emerging from zero (first sample) connecting to both maximum and minimum oocyst counts (second sample; Figure 2b).

**TABLE 1:** Repeatability across sample types—first/second & second/third for Mini‐FLOTAC and McMaster techniques—and across techniques—using the second and third sample—for *S. amaculata* and *Isospora* count and prevalence. The R score lies on a 0–1 scale with a corresponding standard error and confidence interval. Field sample is also referred to as the first sample (1st) and the captive samples are named as the second (2nd) and third (3rd) samples. For details on model structures, see Appendix 1: Table A1.

Model		Parasite	Repeatability between samples	Data	Technique					
					Mini‐FLOTAC			McMaster		
					*R*	SE	95% CI	*R*	SE	95% CI
1a	i, ii	*S. amaculata*	1st–2nd	Count	0.62	0.16	0.26, 0.85	0.91	0.03	0.83, 0.96
	iii, iv		2nd–3rd	Count	0.82	0.05	0.71, 0.91	0.87	0.04	0.77, 0.92
	iii, iv			Prevalence	0.94	0.02	0.96, 0.99	0.97	0.03	0.98, 0.99
1b	i, ii	*Isospora* sp.	1st–2nd	Count	0.12	0.16	0, 0.49	0.11	0.15	0, 0.49
	iii, iv		2nd–3rd	Count	0.62	0.09	0.39, 0.77	0.61	0.10	0.41, 0.77
	iii, iv			Prevalence	0.47	0.19	0.05, 0.74	0.53	0.18	0.09, 0.72

			Repeatability across techniques	Data	Mini‐FLOTAC/McMaster		
					*R*	SE	95% CI
2a	i.	*S. amaculata*	2nd sample	Count	0.93	0.02	0.88, 0.96
	i.			Prevalence	0.98	0.01	0.98, 0.99
	ii		3rd sample	Count	0.93	0.02	0.87, 0.96
	ii			Prevalence	0.98	0.01	0.98, 0.99
2b	i	*Isospora* sp.	2nd sample	Count	0.91	0.03	0.84, 0.94
	i.			Prevalence	0.70	0.21	0.29, 0.99
	ii		3rd sample	Count	0.92	0.03	0.85, 0.95
	ii			Prevalence	0.85	0.15	0.52, 0.99

#### 3.1.1 Repeatability between sample types per technique

Comparing sample estimates of repeatability per technique, we found that estimates of *S. amaculata* count and prevalence were generally very repeatable (Table 1, models 1a i‐iv). We observed that *Isospora* oocysts were not repeatable between the first and second samples in both techniques indicated by very low R values (Table 1, models 1b i, ii). However, between captive samples, repeatability was high in both techniques but slightly lower for *Isospora* prevalence (Table 1, models 1b iii, iv).

#### 3.1.2 Repeatability across techniques

**FIGURE 3:**
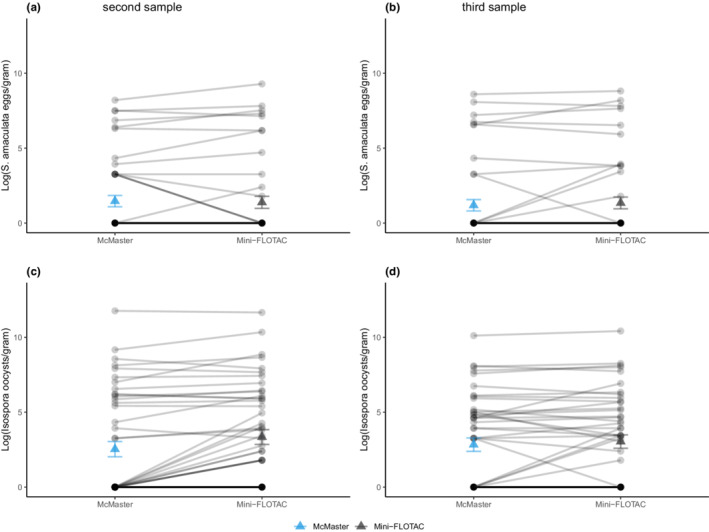
Individual parasite loads compared between the McMaster (blue) and mini‐FLOTAC (black) techniques in captive samples. The data were log‐transformed for (a) second and (b) third samples counted for *S. amaculata* eggs; (c) second and (d) third samples counted for *Isospora* oocysts. Mean parasite counts and standard errors are indicated by triangular points.

When evaluating repeatability within the same sample (comparing estimates from the same sample across techniques, using only captive samples), we found very high repeatability across techniques for both *S. amaculata* eggs and *Isospora* oocysts (Table 1, models 2a & b; Figure 3), suggesting that either method can yield similarly robust results. However, the portion of each sample processed with the McMaster technique appeared to be less sensitive for lower infection loads, especially when estimating *Isospora* infections (Figure 3c,d). We also observed a lower sensitivity at detecting *S. amaculata* eggs in the third sample when using the McMaster technique (Figure 3b). The field samples were not included here, as each individual could only be analyzed with one or the other technique.

### 3.2 What is the probability of detecting false negatives across sample types and techniques?

Overall, we found a higher percentage of false‐negative detections for the field samples (70%) than captive samples (41%; Table 2). These false negatives in field samples were even more obvious for *Isospora* oocysts (81%) than *S. amaculata* eggs (59%). The captive (second and third) samples had a similar percentage of false negatives for both parasite types, though *Isospora* oocysts had a slightly lower percentage (second: 36%, third: 31%) when compared to *S. amaculata* eggs (second: 44%, third: 53%). For *Isospora* oocyst count, when using the mini‐FLOTAC technique, the field sample had a 49% higher rate of false negatives in comparison to captive samples. Similarly, when the McMaster technique was used, the field samples had a 46% higher rate of false negatives when compared to the captive samples. For *S. amaculata* count, the field sample had a 25% higher rate of false negatives when compared to captive samples with the mini‐FLOTAC. In contrast, when using the McMaster technique, the field sample for *S. amaculata* had a 3% lower rate of false negatives when compared to the captive samples.

**TABLE 2:** Percentage of false‐negative detections between techniques and across sample types for *S. amaculata* eggs and *Isospora* sp*.* oocysts. In parentheses, the number of positive samples missed per the total number of positive samples detected is provided. Highlighted in bold are the samples with fewer false negatives per sample and parasite.

Parasite type	Technique	Field sample	Captive sample	
		First	Second	Third
*S. amaculata*	Mini‐FLOTAC	75% (6/8)	53% (9/17)	**47% (8/17)**
	McMaster	**44% (4/9)**	**35% (6/17)**	59% (10/17)
*Isospora* sp.	Mini‐FLOTAC	**74% (14/19)**	**22% (8/37)**	**27% (10/37)**
	McMaster	89% (16/18)	51% (19/37)	35% (13/37)

### 3.3 What factors can influence the detectability of infection status and load?

**FIGURE 4:**
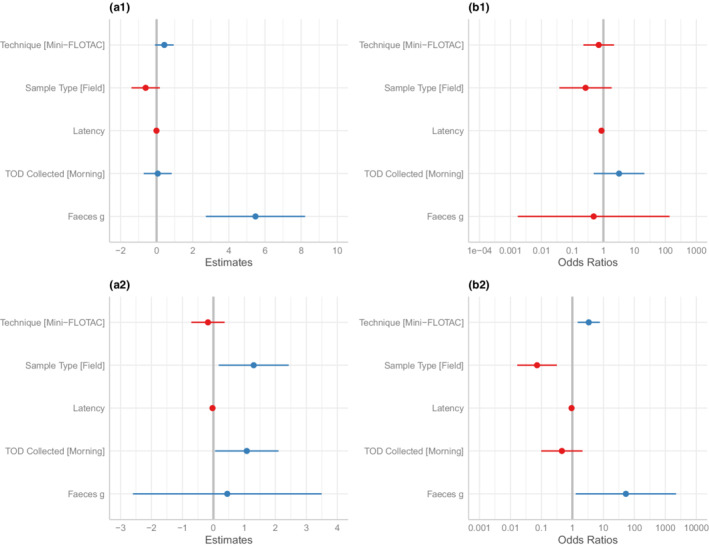
The effect of technique, sample type, latency, time of day (TOD) collected, and feces (g) on parasite counts are represented as model estimates for (a1) *S. amaculata* load and (a2) *Isospora* sp. load. The odds ratio for (b1) the presence of *S. amaculata* and, (b2) the presence of *Isospora* sp. are shown. The gray line is the center at zero, in blue are variables to the right side of the gray line (positive) and in red are variables to the left side (negative) of the gray line. The error bars represent the 95% CI for each variable.

We found that captive samples had a higher probability of detecting *Isospora* oocysts, but recovered lower counts of oocysts when compared with field samples (*β* ± SE = −2.63 ± 0.75, *p* < .01), and this was not observed with *S. amaculata* load (Appendix 1: Table A2, Figure 4). The result for *Isospora* is, however, only based on the loads of six positive field samples, and these are likely to be biased toward samples with high loads (see Figure 2) meaning that this is likely to be a spurious result. We found that the mini‐FLOTAC technique was more likely to detect *Isospora* oocysts (*β* ± SE = 1.22 ± 0.42, *p* < .01), but did not detect higher infection loads (*β* ± SE = −0.18 ± 0.27, *p* = .52).

We further found that latency to process samples had a strong negative effect on the detectability of both *S. amaculata* eggs and *Isospora* oocysts (*β* ± SE = −0.14 ± 0.07, *p* = .03; *β* ± SE = −0.06 ± 0.03, *p* = .01; Figure 4). The probability of detecting a *S. amaculata* infection after 46 days of retrieving the sample is predicted to be as low as 23%, and drop to as little as 1% after 106 days. The probability of detecting *Isospora* infection was affected similarly (Appendix 1: Table A2). The time of the day the sample was collected was identified as a strong contributing factor, with a significant positive effect on morning over afternoon sampling on *Isospora* loads (*β* ± SE = 1.08 ± 0.52, *p* = .04). A very strong effect was also found as feces weight increased, we observed that the infection load for *S. amaculata* also did (*β* ± SE = 5.47 ± 1.36, *p* < .01). Lastly, the random effect—bird ID—contributed a larger variance in the model and this was particularly stronger for *S. amaculata* load (Appendix 1: Table A2).

## 4 DISCUSSION

Our study shows the low sensitivity of field data to fecal sampling for parasites. It suggests that, at least in great tits, a single field sample would rarely provide sufficient data for robust within‐individual estimations of internal parasite loads and prevalence—presence/absence—of infection. We further found several other important factors that warrant considering when planning the sampling design of future studies. For example, the detection of parasites was sensitive to feces weight and processing latency. Another important finding, corroborating previous studies, is that lab techniques are somewhat parasite‐specific, with the mini‐FLOTAC being more sensitive to detecting *Isospora* oocysts and the McMaster marginally better at detecting *S. amaculata* eggs. However, in general, both methods reliably detect the presence and abundance of *Isospora* sp. and *S. amaculata* given sufficiently large samples are collected. Our study confirms the need to carefully consider the potential for sampling variability when collecting samples from the wild, as well as the best technique to use given the parasites of interest to the study.

*Serratospiculoides amaculata* is a characteristic nematode species known to exist as a worm in its adult stage, where the parasite's eggs develop into a male or female adult worm. When these worms aggregate together within the air sac, they reproduce sexually and the female becomes gravid with eggs which are later shed intermittently back into the environment to ensure that transmission to another host is uninterrupted (Van Wettere et al., 2018). *Isospora* sp. is a single‐celled protozoan parasite that reproduces sexually within its host's intestines through cell division producing thousands of oocysts that are passed out through the feces into the environment (Schrenzel et al., 2005). *Isospora* sp. can be transmitted directly through the environment, but *S. amaculata* requires an intermediate host to become infective. Coccidia such as *Isospora* are very widespread and known to infect many passerines. Air sac nematodes such as *S. amaculata*, predominately infecting birds of prey are emerging and becoming common among smaller birds such as passerines (Königová et al., 2013; Martinaud et al., 2009). Therefore, our findings may be applicable to other bird host–parasite systems.

The high rate of false negatives we observed in field samples should urge caution about the informational value of such samples. Field samples usually represent an opportunistic snapshot. By contrast, captive samples pooled the eggs and oocysts shed over a 24‐h period, increasing the chances of detecting infections and minimizing noise. Whether field samples can yield data of sufficient quality needs to be considered in the frame of each biological question. They may not be informative when trying to link parasite status or loads with movement or disease transmission in social networks. Yet as an easy, non‐invasive, and relatively low‐cost method, opportunistic field sampling can certainly be used to monitor populations. *S. amaculata*, for example, was reported for the first time in great tits in a population in Slovakia, less than a decade ago (Königová et al., 2013) and our field samples alone would have extended this parasite's range to southern Germany.

Our analysis of *S. amaculata* eggs suggests that field samples may be most useful for detecting high infections in individuals. Our data (e.g. Figure 2) suggest that highly parasitized individuals were rarely missed in the field samples. By contrast, when *Isospora* were detected in field samples, they were present in higher loads compared to either captive sample, but infections of highly parasitized individuals were often missed in the field samples. One reason for this may be that there is a spike in oocysts shed in response to acute stress, as was described in capybaras *Hydrochoerus hydrochaeris* (Eberhardt et al., 2013). In addition, heavily parasitized birds tend to have diarrhea associated with the inflammation of the intestinal wall leading to release of more feces (Pearson, 2001). By contrast, *S. amaculata* is a unique nematode in that the worms are situated within the air sac of the bird, meaning that the eggs released by the adult female worm have a longer journey to undertake (air sac → trachea → mouth → alimentary canal → intestines) before ending up in the intestine (Königová et al., 2013). Thus, it might take more time for stress‐induced changes to occur. We note, however, that high average parasite counts from field samples of *Isospora* sp. could be related to low sensitivity arising from collecting smaller fecal samples, with many low‐infected individuals having no parasites detected, thereby reducing the mean when we removed those samples from the calculation. In addition, the circadian rhythm of coccidian species shows a rise in parasite loads across the day. As most of the birds sampled in the field in our study were done in the morning, low or no *Isospora* detections may be expected, except in highly parasitized individuals. Our results highlight how sensitivity of each approach and the circadian rhythm of parasites can introduce unexpected biases—in this case resulting in higher mean estimate of infection intensity among infected individuals.

In general, the McMaster technique was more repeatable than the mini‐FLOTAC, which is consistent with previous studies comparing these techniques on horses (Went et al., 2018) and chickens (Daş et al., 2020; Went et al., 2018). This may be attributed to the centralization of parasite eggs due to the gradient effect created by the slide (Bosco et al., 2014). Daş et al. (2020) also noted that the McMaster technique was less sensitive for nematode eggs, with a great decrease observed at a known number of 50 eggs per gram (EPG) or less. This may be the case because the mini‐FLOTAC disc can take up seven times the volume of fecal suspension that the McMaster slide can. We also detected a higher rate of false negatives for *Isospora* infection by McMaster relative to mini‐FLOTAC. Similar results have been found in a study comparing these techniques in domestic birds and livestock (Alowanou et al., 2021; Lozano et al., 2021; Silva et al., 2013). Some of these studies, however, did not report statistically significant results which may imply that—overall—the two techniques are generally reliable.

The percentage of false negatives gives another representation for detectability. It is important to note that these results may be an underestimate of the true rate of false negatives due to the non‐invasive method used in this study. There is a possibility that birds that did not shed eggs or oocysts were indeed parasitized. High *Isospora* fecundity associated with most coccidia species (Burrell et al., 2020) may be responsible for a lower percentage of false negatives in captive samples, as oocysts will be shed in frequent and regular intervals in spite of unique shedding peaks. In studies where precise detection of a specific parasite is crucial, molecular‐based techniques may be more sensitive at detecting and quantifying parasites, especially with low infection loads (Dacal et al., 2018; Reslova et al., 2021). For either approach—molecular or microscopic—at least some individuals should be sampled repeatedly to provide an estimate of the error rate (which should then be reported). In field samples, however, we recorded very high percentage of false negatives throughout, supporting our conclusions about their limitations.

Overall, our models show that most of the variance is explained by individual differences. This points to other environmental and host biological factors, which are well known to predict parasite status (Santoro et al., 2020). From a purely methodological perspective—the aim of our study—we found that more factors influenced the detectability of *Isospora* oocysts than *S. amaculata* eggs, suggesting that the former is more sensitive to study design decisions than the latter. This could be linked to inconsistent egg shedding over the course of the day. The latency to process samples also had a strong negative effect on both parasites, particularly *S. amaculata*. A previous study in horse nematodes (Crawley et al., 2016) found a significant decline in egg counts after a period of 2 weeks. By contrast, feces weight had a significant positive effect on *S. amaculata* load. More fecal matter may increase the likelihood of shedding the parasite's eggs being coughed up and swallowed by the host. This shows that maintaining high accuracy in parasite counts requires prompt processing times and substantial fecal sample, and that any variation in these factors should be accounted for in statistical models.

Because sample collection in captivity lasted for 2 days, we do not expect birds to have become newly infected or re‐infected. New infections are highly unlikely as the cycle of the parasites requires a minimum of 4 days to several weeks (Abebe & Gugsa, 2018; Samour & Naldo, 2001), and *S. amaculata* requires an intermediate host to ensure transmission and infection (Samour & Naldo, 2001). Further, birds were kept under hygienic conditions, where food and water were replaced on a daily basis, and our study was carried out during autumn when lower temperatures are unsuitable for the development of these parasites. However, any studies that include a captive phase should also consider the chances of the study methodology impacting what is being sampled.

Overall, our study highlights the importance of sampling design and sample processing in generating accurate measures of infection status and parasite loads. For *S. amaculata* and *Isospora*, we recommend great care be taken when considering whether to employ field samples in studies that require fine‐scale infection data (e.g., those conducted at the individual level). This is likely to be particularly important for smaller study animals. Such samples may, however, still be useful in detecting the presence of parasites at a broader scale (e.g., a local subpopulation) and at detecting individuals with high infection loads (although this was somewhat less reliable for high *Isospora* infection). We also recommend the mini‐FLOTAC technique as a more accurate and sensitive technique for the detection of *Isospora* oocysts, although both common lab techniques—McMaster and mini‐FLOTAC—are well suited for detecting and estimating *S. amaculata* loads.

## AUTHOR CONTRIBUTIONS

**Salamatu Abdu:** Conceptualization (equal); data curation (lead); formal analysis (equal); investigation (lead); methodology (lead); project administration (lead); writing – original draft (equal); writing – review and editing (lead). **Micheal Chimento:** Investigation (supporting); writing – review and editing (equal). **Gustavo Alarcón‐Nieto:** Investigation (supporting); writing – review and editing (equal). **Daniel Zúñiga:** Conceptualization (equal); investigation (supporting); methodology (supporting); writing – review and editing (supporting). **Lucy M. Aplin:** Conceptualization (equal); funding acquisition (equal); supervision (equal); writing – review and editing (equal). **Damien Farine:** Conceptualization (equal); funding acquisition (lead); methodology (equal); supervision (equal); writing – original draft (equal); writing – review and editing (equal). **Hanja B. Brandl:** Formal analysis (equal); investigation (equal); methodology (equal); supervision (equal); writing – original draft (equal); writing – review and editing (equal).

## FUNDING INFORMATION

This project was funded by the ERC (European Research Council) under the European Union's Horizon 2020 research and innovation programme (850859) awarded to DRF and a Max Planck Society Research Group Leader Fellowship awarded to LMA. SA was funded by a DAAD (German Academic Exchange Service) scholarship for postgraduate studies (personal ref. no. 91730576). DRF was funded by an Eccellenza Professorship Grant of the Swiss National Science Foundation (grant number PCEFP3_187058), and HBB was funded by the Deutsche Forschungsgemeinschaft Centre of Excellence 2117 “Centre for the Advanced Study of Collective Behaviour” (ID 422037984). MC was funded by the International Max Planck Research School for Organismal Biology.

## CONFLICT OF INTEREST

We declare we have no conflict of interest.

## Data Availability

Data and script are available in Edmond, the data repository of the Max Planck Society via the following link: https://dx.doi.org/10.17617/3.8d.

## APPENDIX 1

### REPEATABILITY TESTS AND GENERALIZED LINEAR MIXED MODELS

This appendix provides additional information on the model structure for repeatability tests and generalized linear mixed models run on our two study parasites.

**TABLE A1:** Statistical models for repeatability tests for *S. amaculata* and *Isospora* sp., run with count data (log‐transformed) and prevalence data (present‐absence) respectively

Model		Variables		Data	
		Response	Predictor	Sample_No	Technique
1a	i.	Log/presence‐absence (*S. amaculata*)	Sample_No + (1|Bird_ID)	First, second	Mini‐FLOTAC
	ii.			First, second	McMaster
	iii.			Second, third	Mini‐FLOTAC
	iv.			Second, third	McMaster
1b	i.	Log/presence‐absence (*Isospora* sp.)		First, second	Mini‐FLOTAC
	ii.			First, second	McMaster
	iii.			Second, third	Mini‐FLOTAC
	iv.			Second, third	McMaster
2a	i.	Log/presence‐absence (*S. amaculata*)	Technique + (1|Bird_ID)	Second	
	ii.			Third	
2b	i.	Log/presence‐absence (*Isospora* sp.)		Second	
	ii.			Third	

**TABLE A2:** Binomial and Gaussian (G)LMMs showing the effect of sample type, technique, latency, weight of faeces and time of day on the detection and load (log‐transformed) of *S. amaculata* eggs and *Isospora* sp. oocysts

Parasite	Variable	Detection probability			Infection load		
		*β*	SE	*p*	*β*	SE	*p*
*S. amaculata*	Intercept	5.24	4.60	0.25	3.56 (34%)	1.86	0.07
	Technique: mini‐FLOTAC	−0.35 (0.70)	0.58	0.55	0.43 (54%)	0.26	0.10
	Sample type: field	−1.32 (0.27)	0.99	0.18	−0.61 (−46%)	0.39	0.13
	Latency	−0.14 (0.87)	0.07	0.03*	−0.01 (−1%)	0.03	0.78
	Time of day: morning	1.17 (3.22)	0.96	0.22	0.06 (6%)	0.38	0.87
	Faeces (g)	−0.72 (0.49)	2.88	0.80	5.47 (23646%)	1.36	<0.01*
	*R* ^2^: model	0.74			0.82		
	*R* ^2^: fixed effects	0.15			−0.03		
	*R* ^2^: random effect	0.59			0.84		
*Isospora* sp.	Intercept	2.66	1.85	0.15	6.93 (1022)	1.47	<0.01*
	Technique: mini−FLOTAC	1.22 (3.39)	0.42	<0.01*	−0.18 (−16%)	0.27	0.52
	Sample type: field	−2.63 (0.07)	0.75	<0.01*	1.30 (267%)	0.57	0.03*
	Latency	−0.06 (0.94)	0.03	0.01*	−0.03 (−3%)	0.02	0.17
	Time of day: morning	−0.78 (0.46)	0.78	0.32	1.08 (194%)	0.52	0.04*
	Faeces (g)	3.98 (53.52)	1.90	0.04*	0.45 (57%)	1.53	0.77
	*R* ^2^: model	0.52			0.69		
	*R* ^2^: fixed effects	0.18			0.11		
	*R* ^2^: random effect	0.34			0.57		

*Note*: Asterisks (*) represent significant *p* values (at *p* < .05). Results are back‐transformed by exponentiating the coefficients. Values in parentheses after the predicted means represent odds ratios in the binomial model (detection probability models) and the difference in percent (exp (*β*)−1)*100 from the factor levels in the intercept in the Gaussian model (infection load models). The variance explained by the full model, fixed effects and random effect are reported as *R* ^2^ values.
